# Alveolar macrophage-derived gVPLA2 promotes ventilator-induced lung injury via the cPLA2/PGE2 pathway

**DOI:** 10.1186/s12890-023-02793-x

**Published:** 2023-12-06

**Authors:** Hanghang Han, Qiuwen Xie, Rongge Shao, Jinju Li, Xueke Du

**Affiliations:** 1 Department of Anesthesiology, The Second Affiliated Hospital of Guangxi Medical University, 166 East University Road, Nanning, Guangxi 530007 China; 2 Guangxi Clinical Research Center for Anesthesiology, Guangxi Engineering Research Center for Tissue & Organ Injury and Repair Medicine, Guangxi Key Laboratory for Basic Science and Prevention of Perioperative Organ Disfunction, Guangxi Medical University Cancer Hospital, Guangxi Medical University Cancer Hospital, Guangxi Medical University Cancer Hospital, Nanning, 530021 China

**Keywords:** Ventilator-induced lung injury, Group V phospholipase A2, Alveolar macrophages, Cytoplasmic phospholipase A2, Prostaglandin E2

## Abstract

**Background:**

Ventilator-induced lung injury (VILI) is a clinical complication of mechanical ventilation observed in patients with acute respiratory distress syndrome. It is characterized by inflammation mediated by inflammatory cells and their secreted mediators.

**Methods:**

To investigate the mechanisms underlying VILI, a C57BL/6J mouse model was induced using high tidal volume (HTV) mechanical ventilation. Mice were pretreated with Clodronate liposomes to deplete alveolar macrophages or administered normal bone marrow-derived macrophages or Group V phospholipase A2 (gVPLA2) intratracheally to inhibit bone marrow-derived macrophages. Lung tissue and bronchoalveolar lavage fluid (BALF) were collected to assess lung injury and measure Ca2 + concentration, gVPLA2, downstream phosphorylated cytoplasmic phospholipase A2 (p-cPLA2), prostaglandin E2 (PGE2), protein expression related to mitochondrial dynamics and mitochondrial damage. Cellular experiments were performed to complement the animal studies.

**Results:**

Depletion of alveolar macrophages attenuated HTV-induced lung injury and reduced gVPLA2 levels in alveolar lavage fluid. Similarly, inhibition of alveolar macrophage-derived gVPLA2 had a similar effect. Activation of the cPLA2/PGE2/Ca2 + pathway in alveolar epithelial cells by gVPLA2 derived from alveolar macrophages led to disturbances in mitochondrial dynamics and mitochondrial dysfunction. The findings from cellular experiments were consistent with those of animal experiments.

**Conclusions:**

HTV mechanical ventilation induces the secretion of gVPLA2 by alveolar macrophages, which activates the cPLA2/PGE2/Ca2 + pathway, resulting in mitochondrial dysfunction. These findings provide insights into the pathogenesis of VILI and may contribute to the development of therapeutic strategies for preventing or treating VILI.

**Supplementary Information:**

The online version contains supplementary material available at 10.1186/s12890-023-02793-x.

## Introduction

Mechanical ventilation is a crucial intervention for respiratory failure in critically ill patients [1]; however, it can lead to ventilator-associated lung injury (VILI) [2, 3], significantly increasing morbidity and mortality [4]. Unfortunately, only treatment methods with unsatisfactory efficacy such as regulation of tidal volume and positive end-expiratory pressure are available [5, 6], necessitating the development of novel therapeutic approaches. Alveolar epithelial cell injury is a primary component of VILI pathogenesis [7, 8] and the secretion of proinflammatory factors by immune cells, including alveolar macrophages and neutrophils, plays a pivotal role in VILI-induced alveolar epithelial cell injury [9–11]. Nevertheless, the precise mechanisms by which these inflammatory factors contribute to alveolar epithelial cell injury remain unclear.

Group V phospholipase A2 (gVPLA2) is a pro-inflammatory enzyme belonging to the secreted phospholipase A2 family, playing a potent paracrine role in the inflammatory process and contributing significantly to tissue damage [12, 13]. Recent research indicates that alveolar macrophages express and release gVPLA2 during pulmonary inflammation, implicating their involvement in the pulmonary inflammatory response [14]. Furthermore, it has been confirmed that upregulation of gVPLA2 exacerbates lung function damage in bacterial induced acute lung injury models [14]. Additionally, gVPLA2 has been found to induce the expression of cytoplasmic phospholipase A2, thereby exerting pro-inflammatory effects [15].

Cytoplasmic phospholipase A2 serves as a signaling molecule involved in the inflammatory response and triggers the production of several proinflammatory factors [16]. Prostaglandin E2 (PGE2), a crucial downstream inflammatory factor produced through cPLA2-mediated hydrolysis of intracellular phospholipids [17], has the ability to increase intracellular calcium ion levels [18, 19]. Imbalance in intracellular calcium ion homeostasis has been identified as a key factor contributing to dysfunctional mitochondrial dynamics and subsequent mitochondrial dysfunction [20, 21]. In turn, mitochondrial dysfunction is believed to play a central role in the development of inflammation [22, 23]. However, there is currently no report on whether gVPLA2 is involved in the occurrence and development of VILI.

In this study, we sought to investigate the role of alveolar macrophage-derived gVPLA2 in VILI. Specifically, we aimed to elucidate whether gVPLA2 influences mitochondrial function through the activation of the cPLA2/PGE2/Ca2 + signaling pathway.

## Materials and methods

### Animal

SPF-grade male C57BL/6J mice were obtained from the Center of Guangxi Medical University, Nanning, China (Certificate No.: SCXK-Gui-2020-0004) and were approved by the Institutional Animal Care and Use Committee of Guangxi Medical University. The mice were housed in specific pathogen-free conditions in cages, provided with autoclaved water and food and 8-12-week-old mice were selected for the experiments.

### Animal model

A total of thirty-six mice were divided into six groups. Following anesthesia with an intraperitoneal injection of sodium pentobarbital (50 mg/kg), the groups were subjected to different protocols. The normal control (CON) group underwent intubation without ventilation, the normal tidal volume (NTV) group was ventilated at a tidal volume of 8 mL/kg for 4 h and the large tidal volume (HTV) group received ventilation with a tidal volume of 20 mL/kg for 4 h. The ventilation parameters, including a respiratory rate set at 80 breaths/minute and a PEEP set at 3 cmH2O, have been unified.

Similar to the previous method [24], clodronate liposomes were prepared by combining phosphatidylserine, cholesterol and phosphatidylcholine at a molar ratio of 1:4:6 in chloroform. The model of alveolar macrophage depletion was constructed by evaporating chloroform at 100 rpm at 40 ° C 24 h before mechanical ventilation and passing the liposome solution through a 200 nm filter and nebulizing it to mice. Constructing a macrophage-depletion combined with high tidal volume (CLOD) group using 20mL/kg tidal volume for 4 h in mice with alveolar macrophage depletion.

On the basis of COLD group, intratracheal instillation of 2× 10^6^ bone marrow-derived macrophages 30 min before mechanical ventilation was identified as the supplemental macrophages (BMDM) group.

Conversely, in order to verify whether it is gVPLA2 secreted by macrophages, we constructed a gVPLA2 inhibitor pretreatment group (VAR) group. Unlike the BMDM group, the VAR group used the antagonist varespladib (90 µ M; GC17203, Glpbio) pre-treatment 2 × 10^6^ Bone marrow-derived macrophages for 24 h were instilled into the trachea 30 min before mechanical ventilation.

### Cell culture and treatment

A549 cells, a human alveolar basal epithelial cell line, were obtained from Guangxi Medical University Affiliated Cancer Hospital. The cells were cultured in Dulbecco’s Modified Eagle’s Medium (DMEM) supplemented with 10% fetal bovine serum (FBS) and 1% penicillin-streptomycin at 37 °C in a humidified atmosphere containing 5% CO2. Prior to treatment, the A549 cells were seeded in appropriate culture vessels and allowed to reach approximately 70–80% confluence.

The cell experiments were divided into three groups: a normal control group, a human recombinant gVPLA2-treated (PLA) group and a dimethyl sulfoxide (DMSO) group. The normal control group received no treatment. The PLA group involved treating A549 cells with human gVPLA2 recombinant protein (dissolved in DMSO) at a concentration of 500 nM [25]; (item no. 10,009,563, Cayman) for 4 h. The DMSO group entailed treating A549 cells with an equivalent amount of DMSO as used in the PLA group.

### Extraction and culture of bone marrow-derived macrophages

Bone marrow-derived macrophage extraction and culture steps were consistent with previous studies [24].

### Hematoxylin-eosin staining (HE)

After routine dewaxing and hydration, hematoxylin eosin staining was performed and the slices were dried and sealed (Please refer to the Supplement Word Material for specific methods).

### Immunohistochemical staining

Lung sections were subjected to staining using the Polink-2 plus® Polymer HRP Detection System following the manufacturer’s instructions. Briefly, sections were incubated with primary antibodies against phosphorylated cytoplasmic phospholipase A2 (p-cPLA2) (1:100) overnight at 4 °C. Subsequently, the sections were incubated with detection reagents, followed by color development using 3,3’-diaminobenzidine. Counterstaining with hematoxylin was performed and the sections were mounted and observed under a light microscope. Immunohistochemical staining scores (IRS) were assessed as previously described [2] using the following formula: IRS = staining intensity (SI) × percentage of positive cells (PP).

### Western blotting

Lung tissue was extracted and cellular proteins were obtained. A 10% separation gel and 4% concentrated gel were prepared and proteins were separated by electrophoresis. The separated proteins were then transferred onto a PVDF membrane. The membrane was incubated overnight at 4 °C on a shaker with primary antibodies against GAPDH (1:1000; 2118 S, Cell Signaling Technology), DRP1 (1:1000; Cell Signaling Technology), OPA1 (1:1000; Abcam) and MFN2 (1:1000; Abcam). Subsequently, an anti-rabbit secondary antibody (1:30000; catalog# 5151, Cell Signaling Technology) was incubated for 1 h at room temperature on a shaker. The membrane was scanned using the Odyssey CLX dual color infrared laser imaging system and ImageJ software (NIH, USA) was used to analyze the grayscale values of the bands. The relative protein expression was calculated as the ratio of the grayscale values of the target protein to the internal reference protein.

### Cellular immunofluorescence

The cells were fixed with 4% paraformaldehyde, permeabilized with 0.2% Triton solution, blocked with 10% goat serum, incubated with p-cPLA2 (1:50; Affinity Biosciences) overnight at 4 °C in the refrigerator and incubated with the corresponding immunofluorescent secondary antibody (anti-rabbit secondary antibody: 1:500; Cell Signaling Technology). The nuclei were stained with DAPI, observed by fluorescence microscopy and photographed.

### Transmission electron microscopy

Lung tissues were fixed, embedded, polymerized, trimmed, sectioned with a Leica UC7 ultrathin sectioning machine, stained with uranyl acetate-lead citrate, observed with a Hitachi H-7650 electron microscope and photographed (Please refer to the Supplement Word Material for specific methods).

### Intracellular calcium ion assay

The cell count was adjusted after preparing the lung tissue into a cell suspension. Fluo-4 AM (2 µM; S1060, Biyuntian, Shanghai, China) working solution was added and incubated for 20 min at 37 °C. The cells were observed under a fluorescence microscope and photographed.Intracellular calcium detection in A549 lung epithelial cells: After cell treatment, Fluo-4 AM and Rhod-2 AM working solutions were added, incubated at 37 °C for 20 min, observed under a fluorescence microscope and photographed.

### ATP assay

After lung tissue was homogenized in PBS, the supernatant was collected and assayed according to the instructions of the ATP assay kit (Biyuntian, Shanghai, China) and the chemiluminescence value of each well was detected by a multifunctional enzyme marker. The assay for cellular experiments was performed as described above (Please refer to the Supplement Word Material for specific methods).

### ELISA

The gVPLA2, TNF-α and IL-6 ELISA kits were purchased from Wuhan Huamei and the PGE2 ELISA kit was purchased from Huayun Biologicals and the assays were performed according to the kit instructions (Please refer to the Supplement Word Material for specific methods).

### Statistical analysis

The data were analyzed using GraphPad Prism 8 (GraphPad Software, LLC, San Diego, CA, USA) and presented as Mean ± SEM. The normality of the data distribution was evaluated using the Shapiro-Wilk test. When comparing multiple groups, one-way ANOVA followed by the Tukey post hoc test was applied. A significance level of P < 0.05 was considered statistically significant.

## Results

### Alveolar macrophage depletion attenuates lung injury in VILI mice

**Fig. 1:**
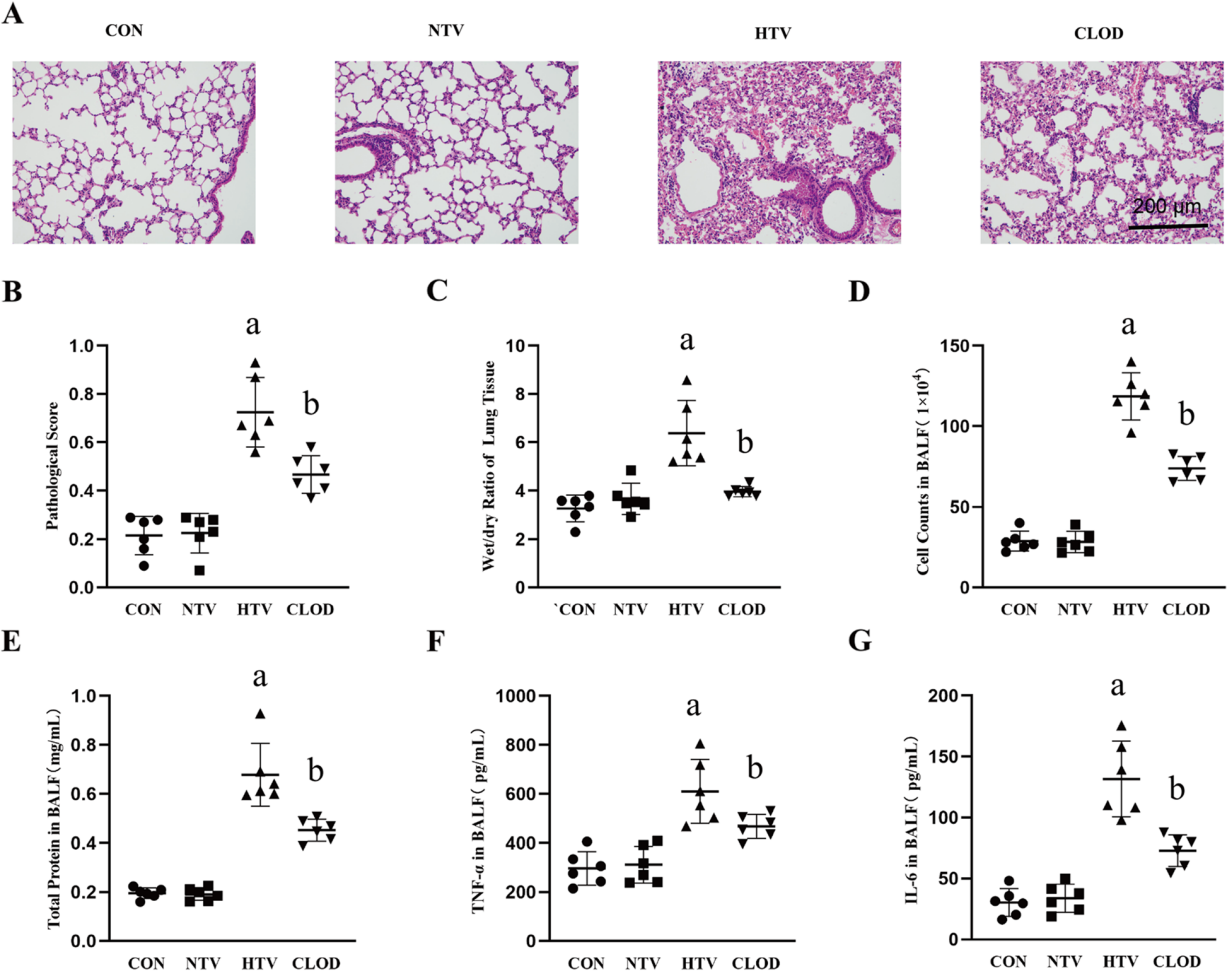
Alveolar macrophage depletion attenuates HTV-induced lung injury and lung inflammation. **A** HE staining for lung histopathological injury; magnification, 200×. **B** Pathology score. **C** The wet/dry ratio of lung tissue. **D** Total cell count in BALF. **E** Total protein content in BALF. **F** TNF-α level in BALF. **G** IL-6 level in BALF. Data are shown as the mean ± SD (*n* = 6). aP < 0.05 vs. CON and NTV group, bP < 0.05 vs. HTV group. CON, blank control; NTV, normal tidal volume; HTV, high tidal volume; CLOD, macrophage-depletion combined with high tidal volume

Building upon our previous investigation on the role of alveolar macrophages in lipopolysaccharide-induced lung injury [24], we sought to examine the impact of alveolar macrophages on VILI. To accomplish this, we evaluated lung injury in mice subjected to high tidal volume ventilation with various controls, including normal tidal volume ventilation and depleted or nondepleted alveolar macrophages. Initially, lung histopathological damage was assessed by performing HE on lung sections. The high tidal volume (HTV) group exhibited significant lung injury, characterized by alveolar septal thickening and other pathological changes when compared to the control (CON) and normal tidal volume (NTV) groups (Fig. 1A-B). Moreover, indicators such as the wet weight/dry weight ratio of lung tissue, total protein content and the number of infiltrating cells in alveolar lavage fluid confirmed the presence of pulmonary edema and increased vascular permeability in the HTV group, consistent with previous studies [26, 27] (Fig. 1C-E). The inflammatory response in lung tissue was assessed by measuring the levels of TNF-α and IL-6 in alveolar lavage fluid, which demonstrated increased levels of these inflammatory factors in the HTV group, corroborating previous findings [28] (Fig. 1F-G).

Subsequently, following the depletion of alveolar macrophages in accordance with established protocols, we observed attenuation of VILI in the mouse model. This was evident from the reduction in lung histopathological damage, pulmonary edema, pulmonary vascular permeability and lung inflammation (Fig. 1A-G).

### Alveolar macrophage depletion decreased gVPLA2 content and downregulated p-cPLA2/PGE2 expression in alveolar lavage fluid of VILI mice

**Fig. 2:**
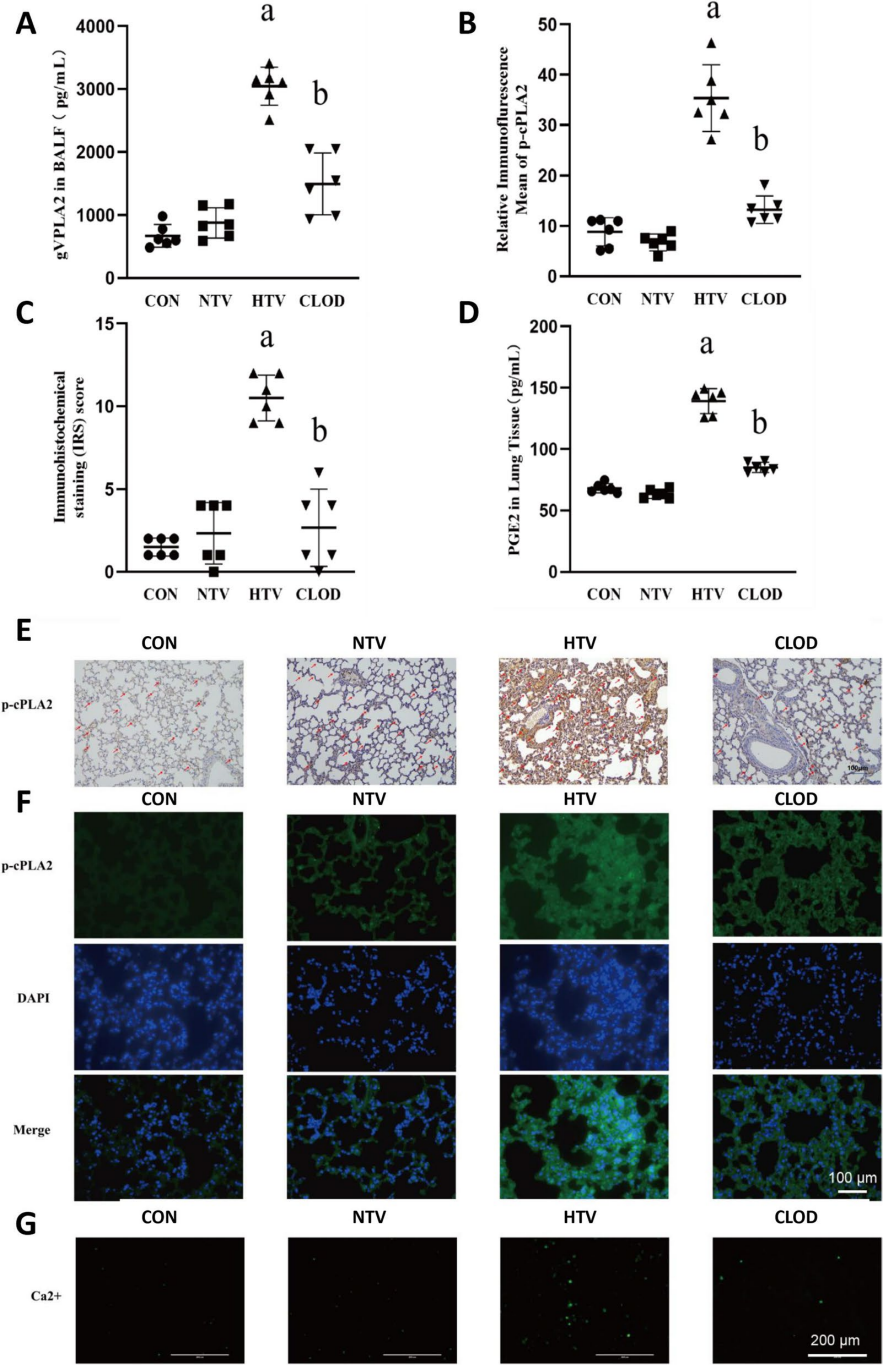
Alveolar macrophage depletion decreased the gVPLA2 content in BALF, downregulated intrapulmonary p-cPLA2/PGE2 and decreased intracellular Ca2+. **A** The gVPLA2 levels in BALF. **B** Relative immunofluorescence mean density of p-cPLA2 in the lung tissue of each group of mice. **C** Immunohistochemical scores of p-cPLA2 in the lung tissue of each group of mice. **D** Levels of PGE2 in lung tissue. **E** Immunohistochemical detection of intrapulmonary p-cPLA2 expression; magnification, 400× (indicated by the red arrow). **F** Immunofluorescence detection of intracellular p-cPLA2 expression in lung, magnification, 400×. **G** Intracellular calcium ion content in single cell suspensions of lung tissue by Fluo-4AM, magnification, 200×. Data are shown as the mean ± SD (*n* = 6). aP < 0.05 vs. CON and NTV group, bP < 0.05 vs. HTV group. CON, blank control; NTV, normal tidal volume; HTV, high tidal volume; CLOD, macrophage-depletion combined with high tidal volume

gVPLA2 is a class of proinflammatory enzymes and a clinical study has demonstrated increased expression of gVPLA2 in alveolar lavage fluid from patients with acute respiratory distress syndrome [29]. Furthermore, the effect of alveolar macrophages on gVPLA2 during lung inflammation is unclear. Therefore, our investigation aimed to determine changes in gVPLA2 levels in the alveolar lavage fluid of VILI mice following macrophage depletion. We observed an increase in gVPLA2 content in the alveolar lavage fluid of the HTV group, which was significantly decreased in the alveolar lavage fluid of the macrophage-depletion combined with high tidal volume (CLOD) group (Fig. 2A).

To assess whether the alterations in gVPLA2 levels in the alveolar lavage fluid of the VILI mouse model affect the activation of its downstream pathway proteins, p-cPLA2 and PGE2, we examined the expression of p-cPLA2 and PGE2. The HTV group demonstrated significant upregulation of p-cPLA2 and PGE2 expression in the lungs. Conversely, alveolar macrophage depletion significantly reduced the expression of p-cPLA2 and PGE2 (Fig. 2B-F). At the same time, we found that the Ca2 + content that regulates mitochondrial function also changes with p-cPLA2 and PGE2 (Fig. 2G).

### Inhibition of alveolar macrophage gVPLA2 downregulates intracellular p-cPLA2/PGE2 protein and reduces intracellular Ca2 + in the lungs of VILI mice

**Fig. 3:**
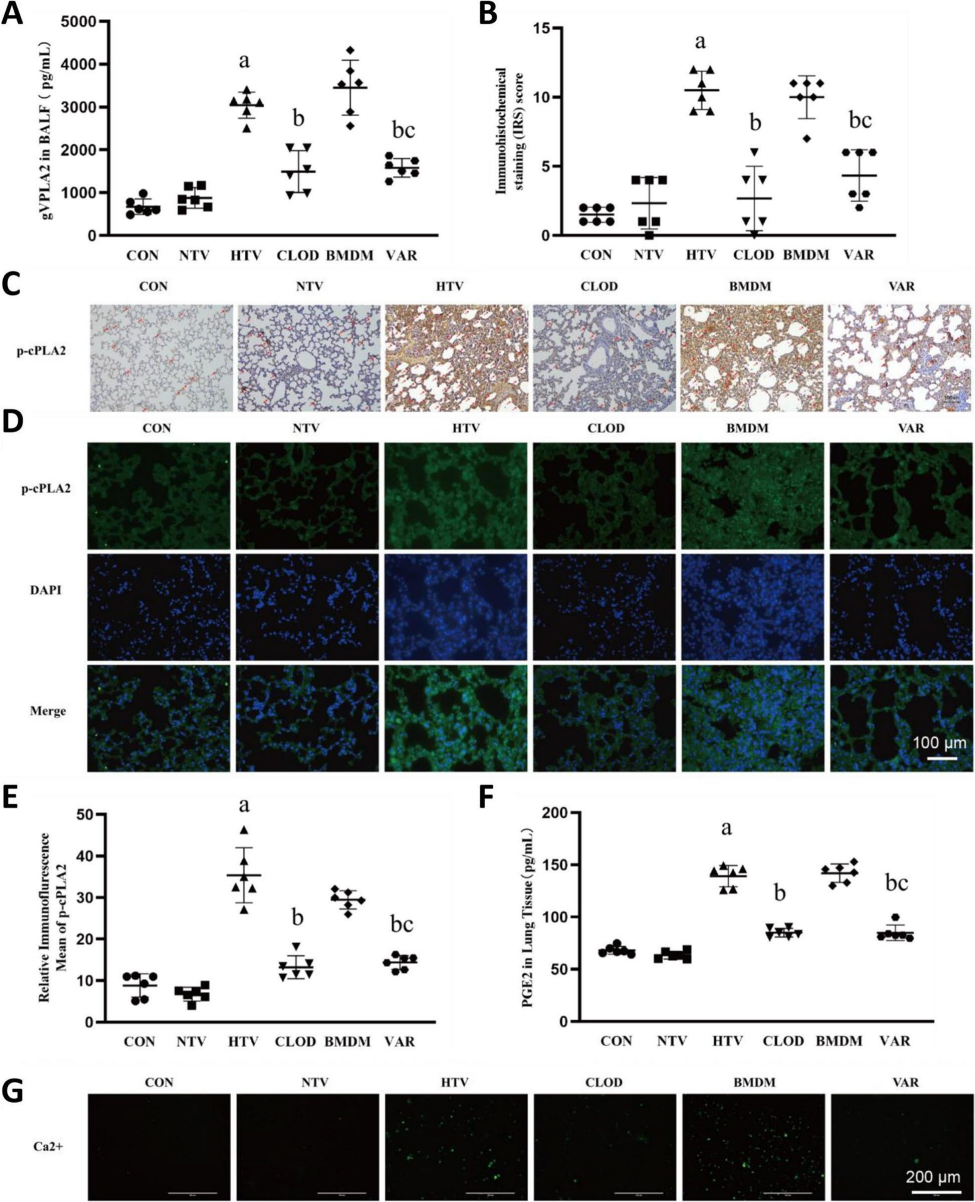
Inhibition of alveolar macrophage gVPLA2 decreased the level of gVPLA2 in alveolar lavage fluid, downregulated intracellular cPLA2/PGE2 and reduced intracellular Ca2+. **A** Levels of gVPLA2 in BALF. **B** Immunohistochemical scores of p-cPLA2 in lung tissue. **C** Immunohistochemical detection of intracellular cPLA2 expression in lungs, magnification, 400× (indicated by the red arrow). **D** Immunofluorescence detection of intracellular p-cPLA2 expression in lung, magnification, 400×. **E** Relative immunofluorescence mean density of p-cPLA2 in lung tissue. **F** Levels of PGE2 in lung tissue. **G** Intracellular calcium ion content in single cell suspensions of lung tissue by Fluo-4AM, magnification, 200×. Data are shown the mean ± SD (*n* = 6). aP < 0.05 vs. CON and NTV group, bP < 0.05 vs. HTV group, cP < 0.05 vs. BMDM group. CON, blank control; NTV, normal tidal volume; HTV, large tidal volume; CLOD, macrophage-depletion combined with high tidal volume; BMDM, alveolar macrophage depletion, followed by intratracheal drip of normal bone marrow-derived macrophages in the high tidal volum; VAR, alveolar macrophage depletion, followed by intratracheal drip of Group V phospholipase A2 inhibitor pretreated bone marrow-derived macrophages in the high tidal volume

Varespladib, a paninhibitor of sPLA2, has been shown to effectively inhibit gVPLA2 activity. In this study, we investigated whether inhibition of gVPLA2 in alveolar macrophages could impact intrapulmonary cPLA2 activation. To address this, we compared intratracheal administration of normal bone marrow-derived macrophages (BMDMs) or BMDMs treated with a gVPLA2 inhibitor in VILI mice. We first measured the expression levels of gVPLA2 in the BALF and as expected, the levels of gVPLA2 were decreased in the CLOD and VAR groups while they were elevated in the BMDM group (Fig. 3A). This also confirmed the successful establishment of the models. Immunofluorescence and immunohistochemistry of lung sections revealed a decrease in p-cPLA2 expression in VAR group (Fig. 3B-E). We also assessed the expression of PGE2 in lung tissue. As expected, PGE2 expression increased in the HTV and alveolar macrophage depletion, followed by intratracheal drip of normal bone marrow-derived macrophages in the high tidal volum (BMDM) groups, while its expression decreased in the CLOD and VAR groups (Fig. 3F). Previous studies have shown that PGE2 can induce intracellular Ca2 + elevation [30]. In our study, we confirmed that intracellular Ca2 + levels increased in the HTV and BMDM groups but decreased in the VAR group (Fig. 3G).

### Inhibition of alveolar macrophage gVPLA2 ameliorates intrapulmonary mitochondrial dynamics disorder and mitochondrial dysfunction in VILI mice

**Fig. 4:**
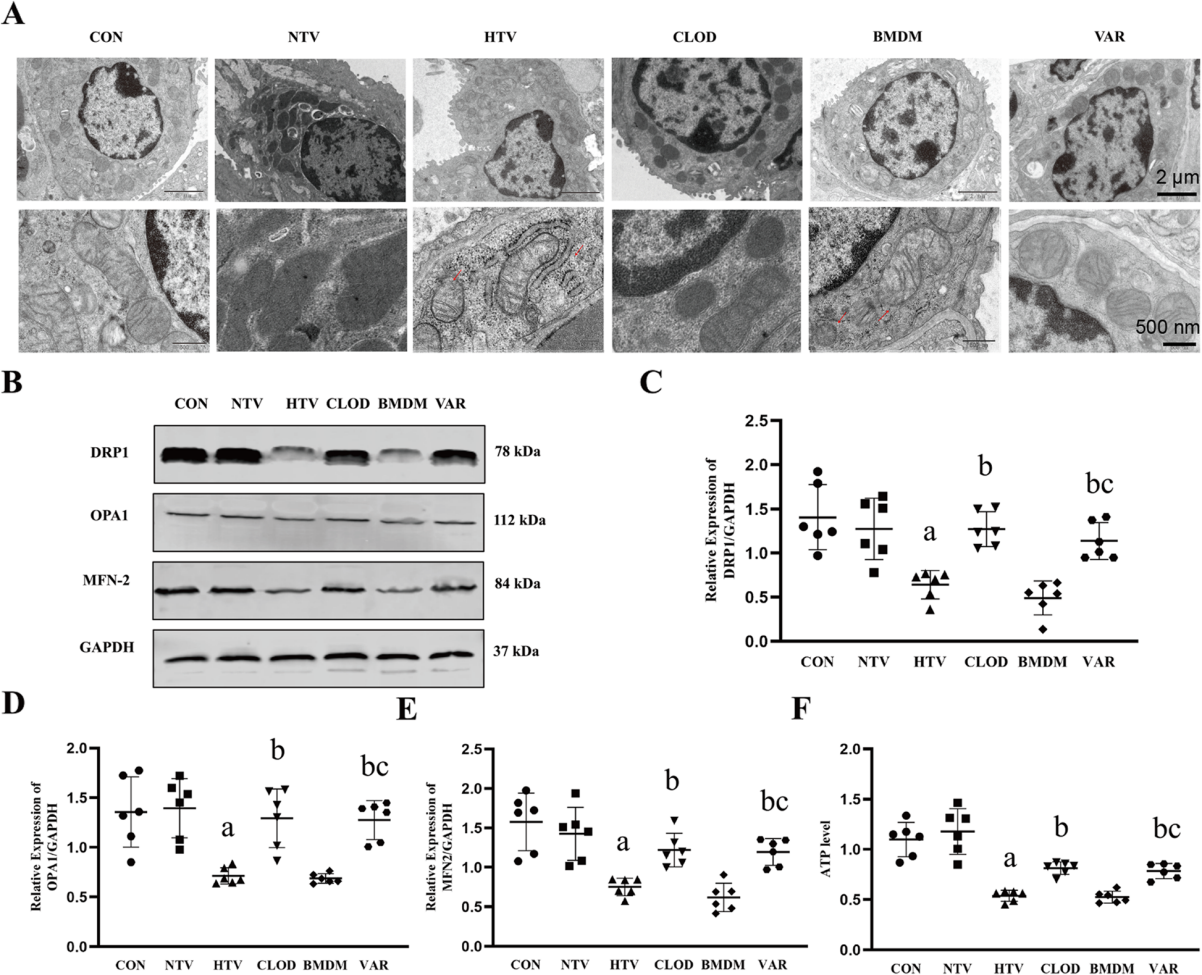
Inhibition of alveolar macrophage gVPLA2 ameliorates mitochondrial kinetic disorders and mitochondrial dysfunction. **A** Transmission electron microscopic observation of structural and morphological changes of mitochondria in alveolar epithelial cells, scale bar, 2 μm(top), 500 nm(bottom). **B** Protein immunoblotting to detect the expression of the mitochondrial splitting protein DRP1 and the mitochondrial fusion proteins OPA1 and MFN2. **C**-**E** The relative expression of DRP1, OPA1 and MFN2 in the lungs of each group of mice using GAPDH as the internal reference protein. **F** The levels of ATP. Data are shown as the mean ± SD (*n* = 6). aP < 0.05 vs. CON and NTV group, bP < 0.05 vs. HTV group, cP < 0.05 vs. BMDM group. CON, blank control; NTV, normal tidal volume; HTV, large tidal volume; CLOD, macrophage-depletion combined with high tidal volume; BMDM, alveolar macrophage depletion, followed by intratracheal drip of normal bone marrow-derived macrophages in the high tidal volum; VAR, alveolar macrophage depletion, followed by intratracheal drip of Group V phospholipase A2 inhibitor pretreated bone marrow-derived macrophages in the high tidal volume

Intracellular calcium overload has been associated with disturbances in mitochondrial dynamics and function [31]. To further assess mitochondrial dysfunction, transmission electron microscopy was performed [32]. The HTV and BMDM groups exhibited obvious mitochondrial damage, characterized by reduced mitochondrial density, loss of mitochondrial cristae, incomplete mitochondrial membranes (indicated by red arrows). On the contrary, the CLOD and VAR groups exhibited less mitochondrial damage during the VILI process compared to the HTV and BMDM groups (Fig. 4A). Meanwhile, we examined the changes in mitochondrial dynamics-related proteins. Western blot analysis revealed decreased expression of the mitochondrial fission protein DRP1, as well as the mitochondrial fusion proteins OPA1 and MFN2, in the HTV and BMDM groups. In contrast, their expression increased in the CLOD and VAR groups (Fig. 4B-E) (Full-length blots are presented in Supplementary Figs. 1, 2, 3, 4). Not surprisingly, the changes in ATP levels, which also reflect mitochondrial function, are consistent with the above indications (Fig. 4F). In conclusion, this demonstrates that the inhibition of gVPLA2 in alveolar macrophages rescued the mitochondrial dynamics and functional impairments in the CLOD and VAR groups.

### Human recombinant gVPLA2 stimulation upregulates p-cPLA2/PGE2 protein and increases intracellular Ca2 + in A549 lung epithelial cells

**Fig. 5:**
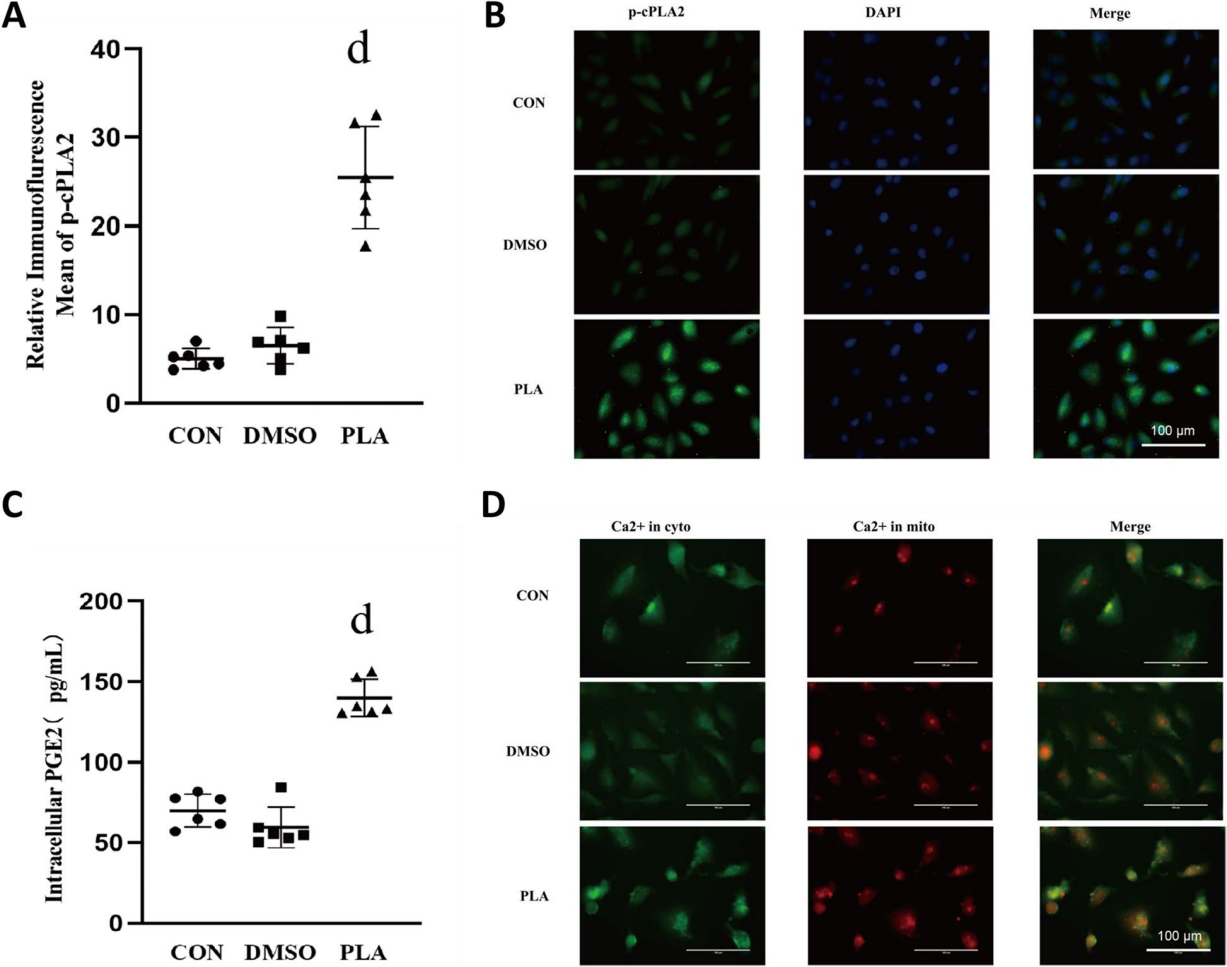
Human recombinant gVPLA2 upregulates p-cPLA2/PGE2 in A549 cells and increases intracellular Ca2+. **A** Relative immunofluorescence indicates the density of intracellular p-cPLA2 in different groups. **B** Immunofluorescence detection of intracellular p-cPLA2 expression in A549 cells, magnification, 400×. **C** Intracellular PGE2 levels in different groups. **D** Detection of cytoplasmic calcium ions in A549 cells by Fluo-4 AM (green light) and intracellular mitochondrial calcium content in A549 cells by Rhod-2 AM (red light), magnification, 400×. Data are shown the mean ± SD (*n* = 6). dP < 0.05 vs. CON and DMSO groups. CON, blank control; DMSO, dimethyl sulfoxide solvent control; PLA, human recombinant gVPLA2-treated

To further validate the impact of gVPLA2 on the p-cPLA2/PGE2 pathway, we conducted in vitro experiments using A549 lung epithelial cells. Following treatment with human recombinant gVPLA2, intracellular p-cPLA2 expression was assessed by cellular immunofluorescence, revealing an increase in p-cPLA2 levels compared to the CON and dimethyl sulfoxide solvent control (DMSO) groups (Fig. 5A-B). Consistent with our previous findings, PGE2 expression was elevated in the PLA group (Fig. 5C). Calcium overload was observed in both the cytoplasm and mitochondria of the PLA2 group, as indicated by Fluo-4 AM and Rhod-2 AM dye labeling of intracellular Ca2+ (Fig. 5D).

### Human recombinant gVPLA2 stimulation causes mitochondrial dynamics disorder and mitochondrial dysfunction in A549 lung epithelial cells

**Fig. 6:**
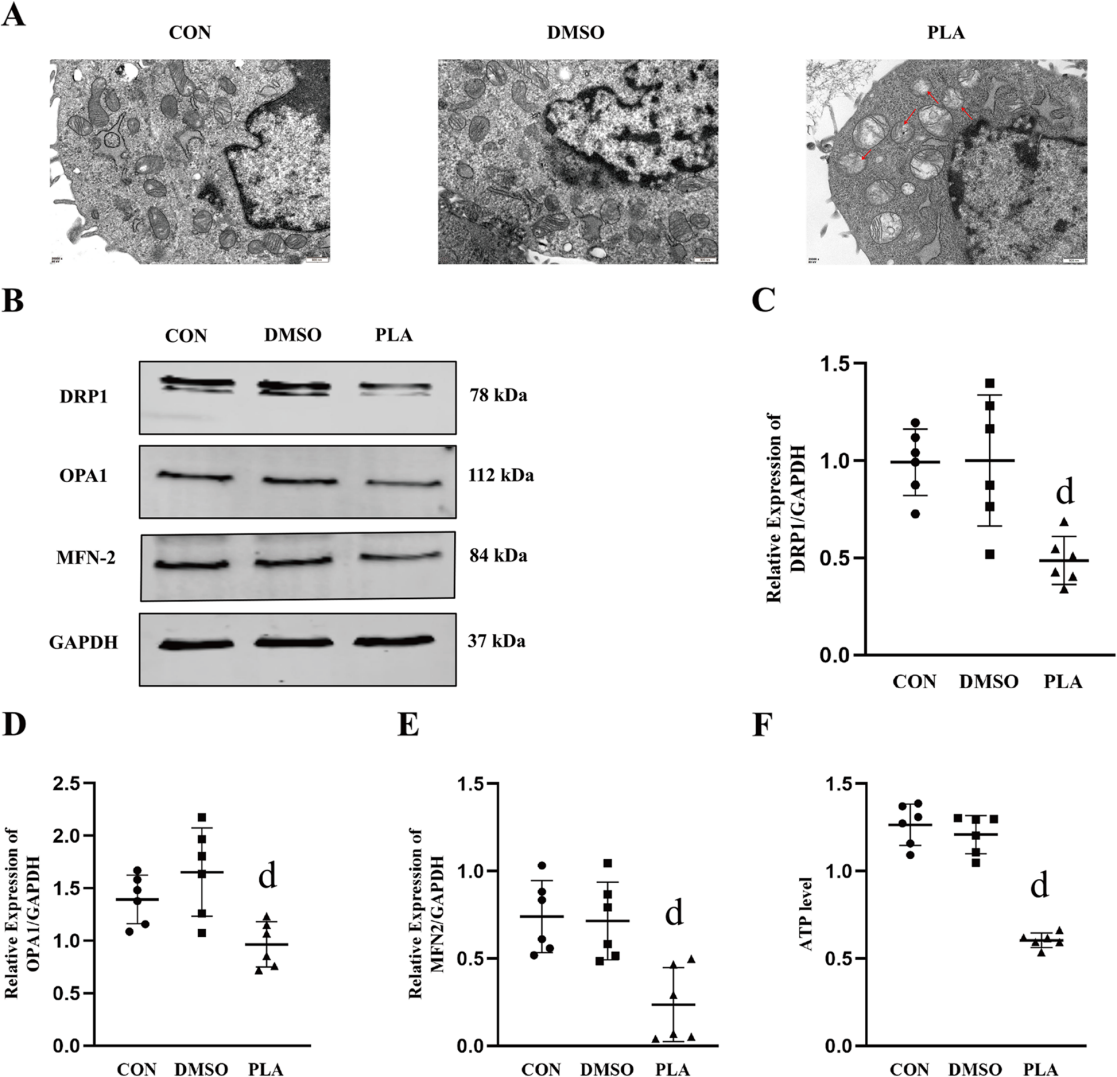
Human recombinant gVPLA2 induces mitochondrial dynamics disorder and mitochondrial dysfunction in A549 cells. **A** Transmission electron microscopy was used to observe the changes in mitochondrial structure and morphology in different groups of cells; scale bar, 500 nm. **B** The expression of the mitochondrial fission protein DRP1 and the mitochondrial fusion proteins OPA1 and MFN2. **C**-**E** The relative expression of DRP1, OPA1 and MFN2 using GAPDH as the internal reference protein. **F** The levels of ATP. Data are shown the mean ± SD (*n* = 6). dP < 0.05 vs. CON and DMSO groups. CON, blank control; DMSO,dimethyl sulfoxide solvent control; PLA, human recombinant gVPLA2-treated

To confirm whether exogenous gVPLA2 can induce mitochondrial damage, we examined the effect of human recombinant gVPLA2 on mitochondria in A549 lung epithelial cells using transmission electron microscopy. We observed mitochondrial injury features, such as reduced mitochondrial density and the disappearance of mitochondrial cristae, in A549 cells (indicated by the red arrow) after treatment with human recombinant gVPLA2 (Fig. 6A).

Furthermore, we confirmed the impact of human recombinant gVPLA2 on mitochondrial dynamics-related proteins in A549 cells. As anticipated, western blot analysis demonstrated a decrease in DRP1, OPA1 and MFN2 expression in A549 cells after treatment with human recombinant gVPLA2 (Fig. 6B-E) (Full-length blots are presented in Supplementary Figs. 5, 6, 7, 8). ATP production, a critical mitochondrial function, was also assessed in A549 cells and found to be reduced following treatment with human recombinant gVPLA2 (Fig. 6F).

### Inhibition of alveolar macrophage gVPLA2 attenuates lung injury in VILI mice

**Fig. 7:**
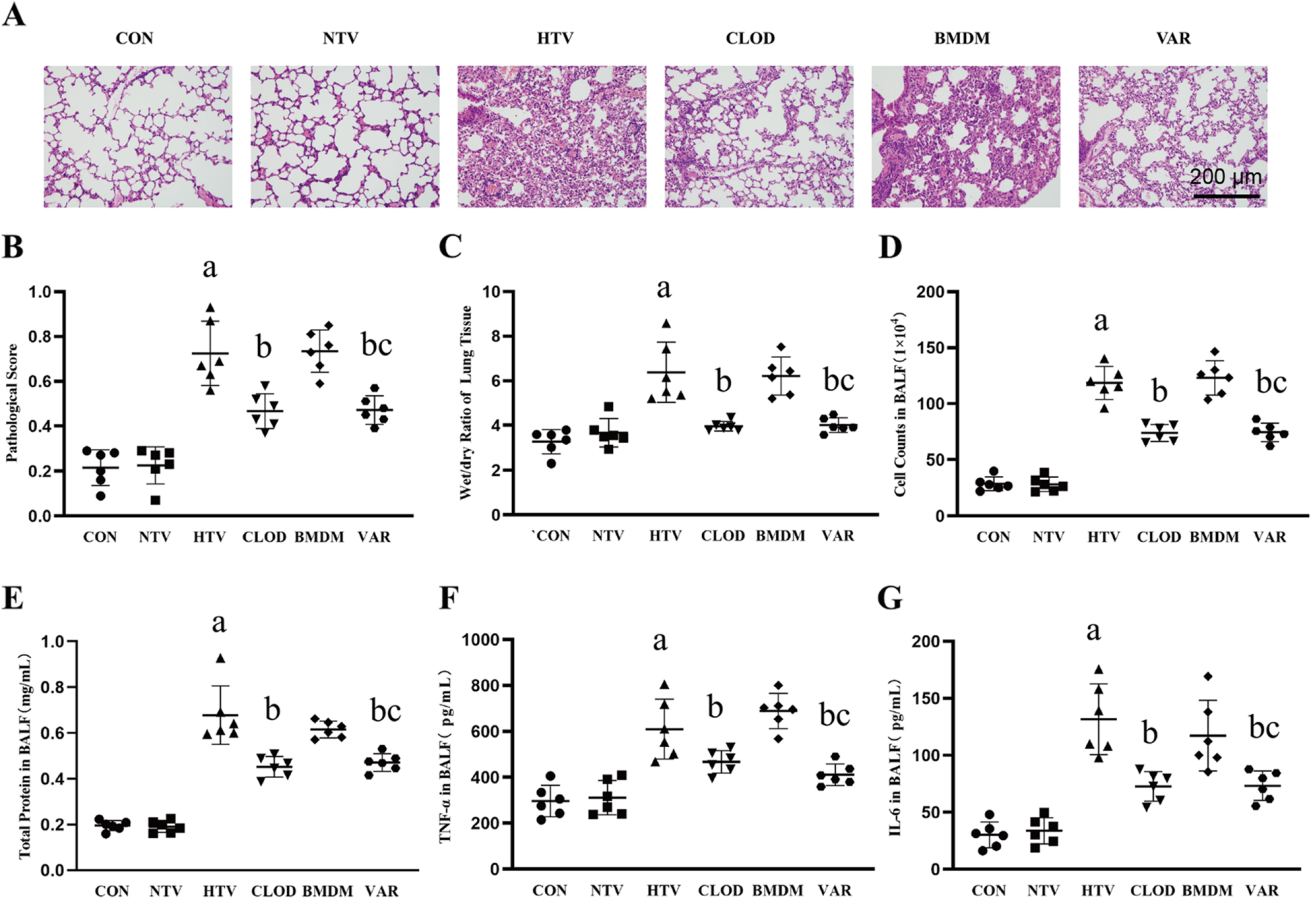
Inhibition of alveolar macrophage gVPLA2 attenuates lung injury and the inflammatory response. **A** HE staining for lung histopathological injury; magnification, 200×. **B **Pathology score. **C** The wet/dry ratio of lung tissue. **D** Total cell count in BALF. **E** Total protein content in BALF. **F** TNF-α level in BALF. **G** IL-6 level in BALF. Data are shown as the mean ± SD (*n* = 6). aP < 0.05 vs. CON and NTV group, bP < 0.05 vs. HTV group, cP < 0.05 vs. BMDM group. CON, blank control; NTV, normal tidal volume; HTV, large tidal volume; CLOD, macrophage-depletion combined with high tidal volume; BMDM, alveolar macrophage depletion, followed by intratracheal drip of normal bone marrow-derived macrophages in the high tidal volum; VAR, alveolar macrophage depletion, followed by intratracheal drip of Group V phospholipase A2 inhibitor pretreated bone marrow-derived macrophages in the high tidal volume

Finally, to assess the impact of alveolar macrophage-derived gVPLA2 on lung injury in VILI mice, we examined whether inhibition of alveolar macrophage gVPLA2 could alter lung histopathological changes. As expected, the pathological evaluation of lung tissue sections revealed a reduction in lung histopathological damage in VILI mice following inhibition of alveolar macrophage gVPLA2. This was evident through the absence of alveolar septum thickening and other relevant indicators (Fig. 7A-B). Similarly, compared to the HTV and BMDM groups, inhibition of alveolar macrophage gVPLA2 led to decreased lung tissue wet-to-dry (W/D) ratios, total cell counts, total protein levels and levels of the inflammatory factors TNF-α and IL-6 in the alveolar lavage fluid of VILI mice (Fig. 7C-G). Hence, inhibition of alveolar macrophage gVPLA2 demonstrated a protective effect against lung injury in VILI mice.

## Discussion

VILI is a serious clinical adverse effect of mechanical ventilation in patients. Previous clinical studies [33, 34] have reported increased mortality in patients with respiratory dysfunction associated with VILI development. Currently, there are no effective treatments available for VILI. Therefore, it is crucial to gain a better understanding of the pathophysiology of VILI and identify potential therapeutic targets. Studies have demonstrated [35, 36] that intrapulmonary infiltration of inflammatory cells, such as alveolar macrophages and neutrophils and the secretion of inflammatory factors play a significant role in the development of VILI. Indeed, Melton et al. identified in their study that one of the mechanisms contributing to VILI due to mechanical ventilation with high tidal volume involves the adverse impact of gVPLA2 on the inflammatory response and barrier function of pulmonary epithelial cells [37]. They concluded that gVPLA2, as a downstream target of pathological mechanical stretching, holds potential physiological significance. Given our prior knowledge that macrophages are capable of secreting gVPLA2 [14, 38], the critical question of whether depletion of gVPLA2 secreted by alveolar macrophages will alleviate VILI remains an urgent issue to be addressed. In our research, we aimed to elucidate the important role of alveolar macrophages in HTV-induced lung injury and the associated inflammatory response by assessing pulmonary edema, lung tissue permeability, histopathological changes and inflammatory factors.

In this study, we provide evidence to support the central role of alveolar macrophage secretion of gVPLA2 in promoting mitochondrial damage during the development of VILI. We observed that alveolar macrophage-derived gVPLA2 induces calcium overload-associated mitochondrial dynamics disorders and mitochondrial dysfunction, leading to the activation of the cPLA2/PGE2 signaling pathway and subsequent alveolar epithelial cell injury. Notably, inhibition of alveolar macrophage gVPLA2 significantly attenuated lung injury in VILI mice, suggesting that gVPLA2 may serve as an effective target for the treatment of VILI and inflammation.

gVPLA2 is expressed and secreted by immune cells such as macrophages and bone marrow-derived mast cells in response to inflammatory stimuli [39, 40]. It functions in a paracrine manner to regulate intracellular cPLA2 activation in neighboring cells during inflammatory cell activation, leading to the hydrolysis of intracellular phospholipids and the release of proinflammatory factors like PGE2 [41–43]. In the context of lung injury, gVPLA2 plays a significant proinflammatory role, as evidenced by studies showing that mice overexpressing gVPLA2 develop severe lung injury and experience high mortality rates [44]. Hence, elevated levels of gVPLA2 are closely associated with the development of lung injury. Therefore, targeting gVPLA2 may hold therapeutic potential for mitigating VILI and inflammation.

Mitochondrial dysfunction has been observed in the context of the inflammatory response [45, 46] and increased intracellular calcium ion concentrations have been shown to have detrimental effects on mitochondria [47, 48]. PGE2 has been demonstrated to participate in the regulation of intracellular calcium ions and to play an active role in immune and inflammatory responses [49–51]. However, it remains unclear whether PGE2 contributes to calcium overload-related mitochondrial damage during VILI, thereby affecting HTV-induced inflammation and lung injury. In our present study, we found that HTV ventilation induced elevated PGE2 expression and increased lung calcium ion levels, along with decreased expression of mitochondrial dynamics-related proteins, mitochondrial damage and reduced ATP production. Importantly, these changes were reversed by inhibiting gVPLA2 secretion from alveolar macrophages. In vitro treatment of lung epithelial cells with human recombinant gVPLA2 further confirmed the roles of gVPLA2 and the cPLA2/PGE2/Ca2 + pathway in mitochondrial dynamics and function. These findings strongly support the notion that alveolar macrophages secrete gVPLA2, which activates the cPLA2/PGE2 pathway, resulting in increased intracellular Ca2 + levels and subsequent disruption of mitochondrial dynamics and function.

Limitations of the study should be acknowledged. First, our study focused on the role of alveolar macrophage-derived gVPLA2 in VILI; however, other cell types and molecular mechanisms may also contribute to the development of VILI. Further investigations are needed to explore the involvement of other cell types and signaling pathways. Second, although we used an established mouse model of VILI, it is important to note that animal models cannot fully recapitulate the complexity of VILI in human patients. Future studies should attempt to validate our findings in clinical settings. Third, while our cellular experiments provided valuable insights into the mechanistic aspects of gVPLA2-mediated mitochondrial dysfunction, it is crucial to confirm these findings using in vivo models or human samples. Finally, although we observed promising results regarding the potential therapeutic role of inhibiting gVPLA2 in VILI, further studies are required to evaluate the long-term effects and the feasibility of targeting gVPLA2 in a clinical setting. Overall, these limitations should be considered when interpreting the results and implications of our study.

## Conclusion

**Fig. 8:**
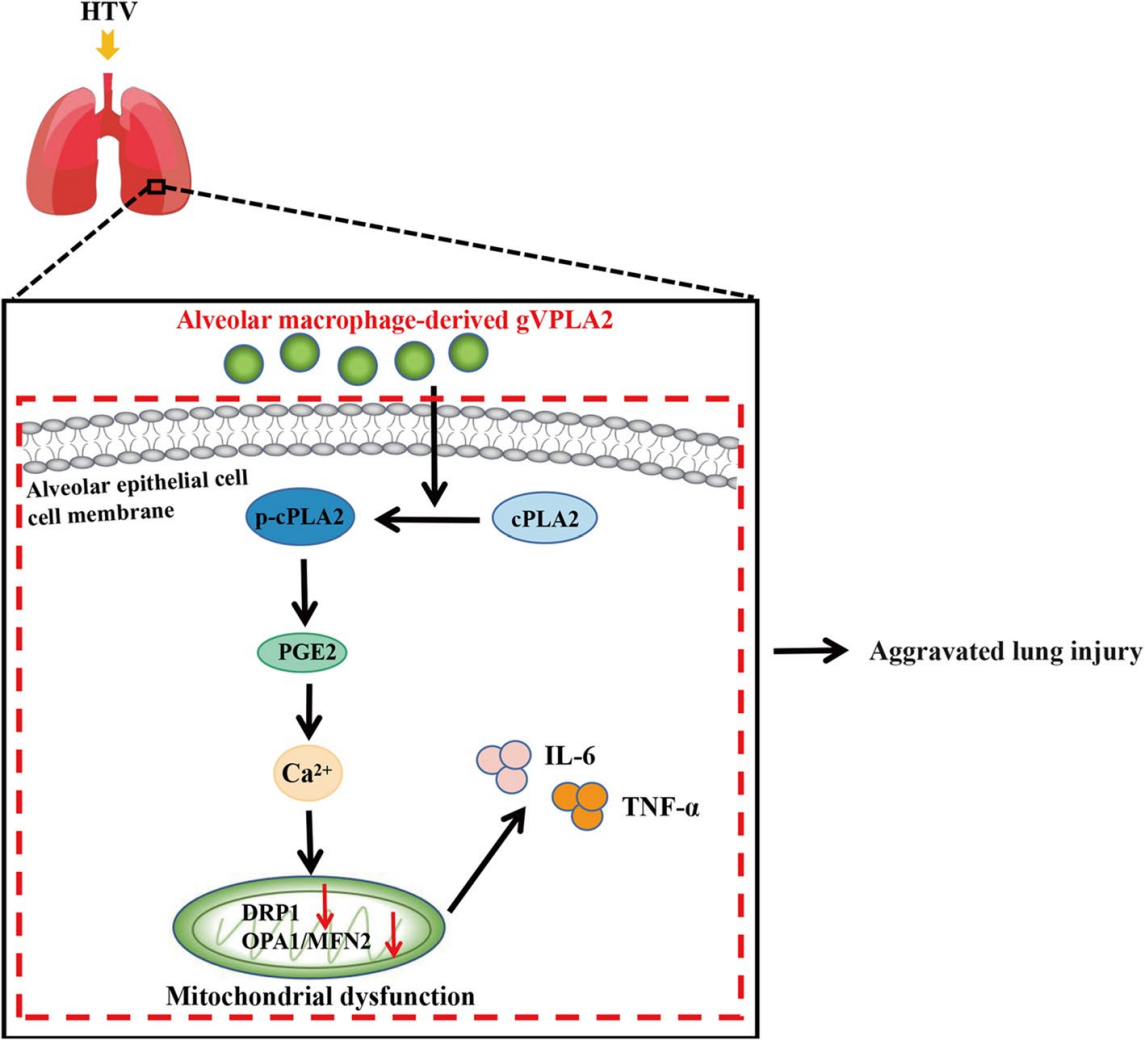
High tidal volume mechanical ventilation promotes gVPLA2 secretion by alveolar macrophages, which in turn activates the intrapulmonary cPLA2/PGE2/Ca2 + pathway, leading to disrupted intracellular mitochondrial dynamics and mitochondrial dysfunction in lung tissue

In conclusion, we identified alveolar macrophage-secreted gVPLA2 as a crucial protein involved in the pathogenesis of VILI in mice and our results demonstrate that gVPLA2 exacerbates lung injury and inflammation VILI mice through the activation of the cPLA2/PGE2 signaling pathway, leading to calcium-mediated mitochondrial damage (Fig. 8). We propose that gVPLA2 represents a potentially effective target for the development of therapeutic interventions for VILI. Furthermore, our findings may have implications for other respiratory diseases that are driven or influenced by gVPLA2, such as bacterial infectious pneumonia [25, 52], allergic pneumonia and asthma [53].

### Supplementary Information

**Additional file 1.**

**Additional file 2.**

## Data Availability

All data generated or analyzed during this study are included in this article. Further enquiries can be directed to the corresponding author.

## Abbreviations

VILI: Ventilator-induced lung injury
HTV: High tidal volume
gVPLA2: Group V phospholipase A2
BALF: Bronchoalveolar lavage fluid
p-cPLA2: Phosphorylated cytoplasmic phospholipase A2
PGE2: Prostaglandin E2
HE: Hematoxylin-eosin staining
IRS: Immunohistochemical staining scores
SI: Staining intensity
PP: Percentage of positive cells
CLOD: Macrophage-depletion combined with high tidal volume
BMDMs: Bone marrow-derived macrophages
VAR: Alveolar macrophage depletion, followed by intratracheal drip of Group V phospholipase A2 inhibitor pretreated bone marrow-derived macrophages in the high tidal volume
BMDM: Alveolar macrophage depletion, followed by intratracheal drip of normal bone marrow-derived macrophages in the high tidal volum
DMSO: Dimethyl sulfoxide solvent control
W/D: Wet-to-dry
