# Supplementary material for: Alveolar macrophage-derived gVPLA2 promotes ventilator-induced lung injury via the cPLA2/PGE2 pathway
Source: BMC Pulm Med. 2023 Dec 6;23:494. doi: 10.1186/s12890-023-02793-x (PMC10701980; doi:10.1186/s12890-023-02793-x)
Supplement: Supplementary file 1 — Additional file 1. [file 12890_2023_2793_MOESM1_ESM.docx]

**Hematoxylin-eosin staining (HE)**

1.Tissue Sampling, Freezing, Sectioning, and Hematoxylin and Eosin (H&E) Staining Process for Mouse Lung Tissue:

2.Tissue Sampling: a. After the mice were sacrificed, the chest cavity was opened, and the lungs were exposed and carefully excised. b. The lung tissue was rinsed with chilled phosphate-buffered saline (PBS) to remove any blood and excess tissues. c. The lung lobes were then carefully dissected and placed in a suitable container for further processing.

3.Freezing: a. The lung tissue samples were immediately transferred to a cryomold filled with optimal cutting temperature (OCT) compound. b. The cryomold was placed on dry ice to rapidly freeze the tissue-embedded OCT for at least 15-30 minutes until completely solidified. c. The frozen tissue-embedded OCT block was then securely stored at -80°C until further processing.

4.Sectioning: a. The frozen tissue-embedded OCT block was equilibrated to the cryostat temperature (-20 to -25°C) for optimal sectioning. b. Using a cryostat, lung tissue sections of desired thickness (typically 5-10 µm) were cut and mounted onto glass microscope slides. c. The tissue sections were allowed to dry thoroughly and then stored at -20°C until staining.

5.Hematoxylin and Eosin (H&E) Staining: a. The lung tissue sections were removed from the freezer and allowed to reach room temperature. b. The tissue sections were immersed in formalin for fixation, followed by washing with running water to remove the OCT compound. c. The sections were then stained with hematoxylin to visualize cell nuclei, followed by eosin to provide contrast and stain the cytoplasm. d. After staining, the sections were dehydrated in a series of alcohol washes, cleared in xylene, and coverslipped with mounting medium.

**Transmission electron microscopy**

1.Tissue Collection and Fixation: a. Following euthanasia, the chest cavity of the mouse was quickly opened, and the lungs were exposed. b. A section of the lung tissue was carefully excised and dissected into small pieces (1 mm³) using a sharp scalpel or scissors. c. The lung tissue was immediately immersed in a pre-cooled fixative such as 2.5% glutaraldehyde in 0.1 M phosphate buffer (pH 7.4) for 2-4 hours.

2.Post-fixation and Processing: a. After fixation, the lung tissue samples were rinsed in buffer to remove excess fixative and then post-fixed in 1% osmium tetroxide for 1-2 hours at room temperature. b. The samples were dehydrated through a series of alcohol solutions (e.g., 50%, 70%, 90%, and 100% ethanol) and then infiltrated and embedded in a resin such as epoxy resin.

3.Sectioning and Staining: a. Ultrathin sections (50-70 nm) of the embedded lung tissue were cut using an ultramicrotome equipped with a diamond knife. b. The sections were collected on copper grids and stained with heavy metals such as uranyl acetate and lead citrate to enhance contrast for TEM imaging.

4.Transmission Electron Microscopy (TEM) Imaging: a. The prepared grids with ultrathin sections were loaded into a transmission electron microscope. b. TEM images of the lung tissue ultrastructure were acquired at various magnifications to visualize subcellular organelles, membranes, and other ultrastructural details.

**ATP assay and ELISA**

1.Tissue Sampling and Preparation: a. Following euthanasia, the chest cavity of the mouse was pened, and the lungs were exposed. b. A portion of the lung tissue was carefully excised and rinsed with ice-cold phosphate-buffered saline (PBS) to remove any blood or debris. c. The lung tissue was blotted dry and weighed to ensure accurate homogenization.

2.Tissue Homogenization: a. The lung tissue sample was minced into smaller pieces using a sterile scalpel or scissors to facilitate homogenization. b. The tissue was transferred to a suitable homogenization vessel and PBS was added at a ratio of 100 mg tissue per 1 mL of PBS. c. The tissue-PBS mixture was homogenized using a tissue homogenizer homogenizer until a uniform tissue suspension was obtained.

3.Centrifugation and Collection of Supernatant: a. The homogenized tissue suspension was centrifuged at a low speed (1000 g) for 15 minutes at 4°C to pellet any debris or unbroken cells. b. The supernatant, containing soluble proteins and cellular components, was carefully collected and transferred to a clean microcentrifuge tube.

4.Protein Storage: The homogenate was aliquoted into microcentrifuge tubes and used immediately for ATP and ELISA analysis.

5.ATP Assay Monitoring: a. The ATP assay kit protocol was followed, and the fluorescence signal generated was measured using a fluorescence spectrophotometer. b. The ATP concentration in the supernatant was determined based on a standard curve generated with known ATP concentrations and expressed as nanomoles per milligram of protein.

6.ELISA Monitoring: a. The ELISA assay was performed according to the manufacturer's instructions, where the supernatant samples were added to the ELISA plate wells coated with specific capture antibodies. b. The detection antibodies, enzyme conjugates, and substrate solutions were added sequentially, and the colorimetric or chemiluminescent signal was measured using a microplate reader. c. The concentration of the target analyte in the lung tissue supernatant was determined by comparing the optical density values to a standard curve generated with known concentrations of the target analyte.
