# Supplementary material for: Alveolar macrophage-derived gVPLA2 promotes ventilator-induced lung injury via the cPLA2/PGE2 pathway
Source: BMC Pulm Med. 2023 Dec 6;23:494. doi: 10.1186/s12890-023-02793-x (PMC10701980; doi:10.1186/s12890-023-02793-x)
Supplement: Supplementary file 2 — Additional file 2. [file 12890_2023_2793_MOESM2_ESM.pptx]

## Slide 1
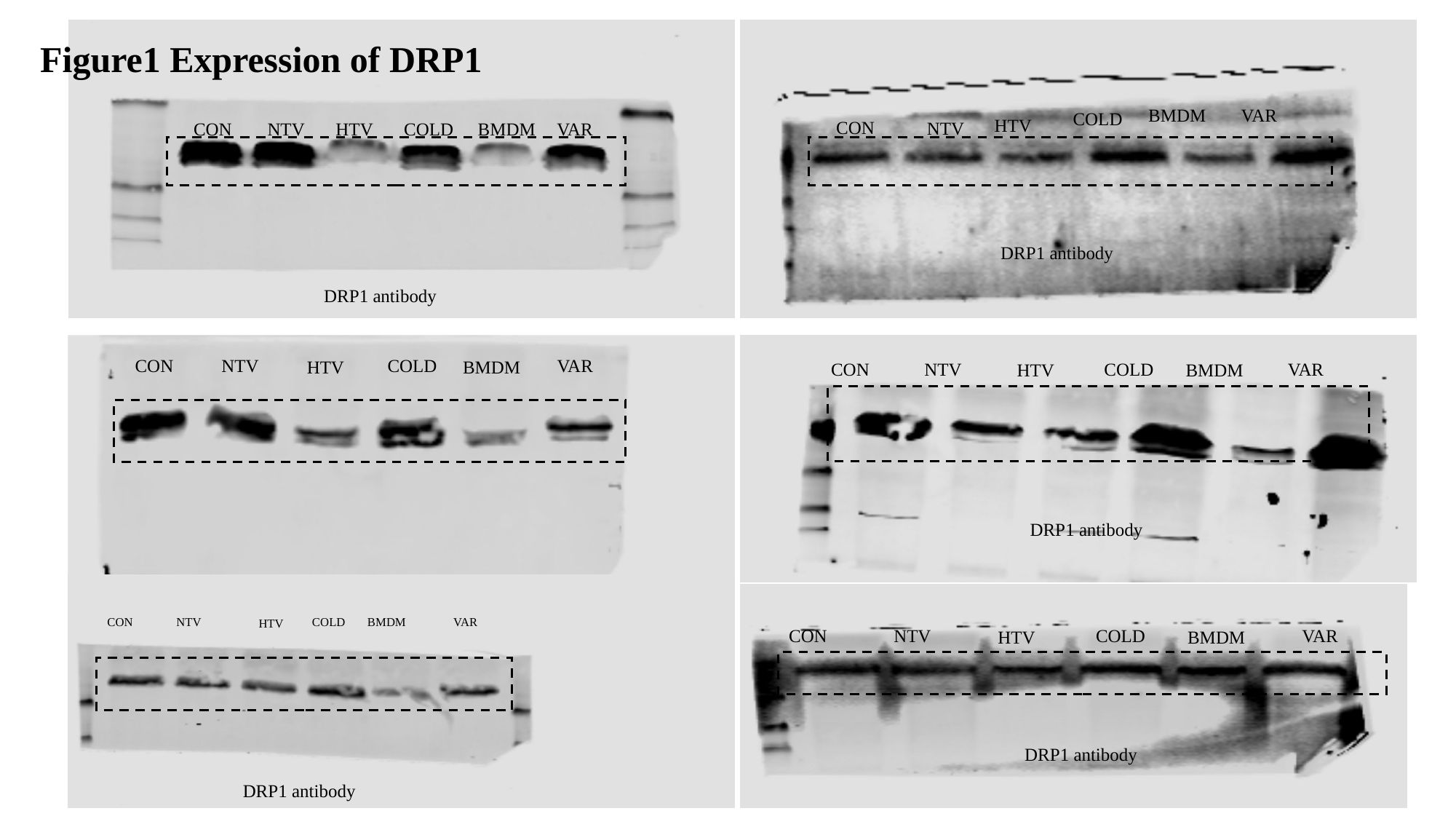

CON
NTV
HTV
COLD
BMDM
VAR
DRP1 antibody
Figure1 Expression of DRP1
VAR
BMDM
COLD
HTV
CON
NTV
DRP1 antibody
CON
NTV
COLD
VAR
HTV
BMDM
DRP1 antibody
CON
NTV
COLD
VAR
HTV
BMDM
DRP1 antibody
CON
NTV
COLD
BMDM
VAR
HTV
DRP1 antibody
CON
NTV
COLD
VAR
HTV
BMDM
DRP1 antibody

## Slide 2
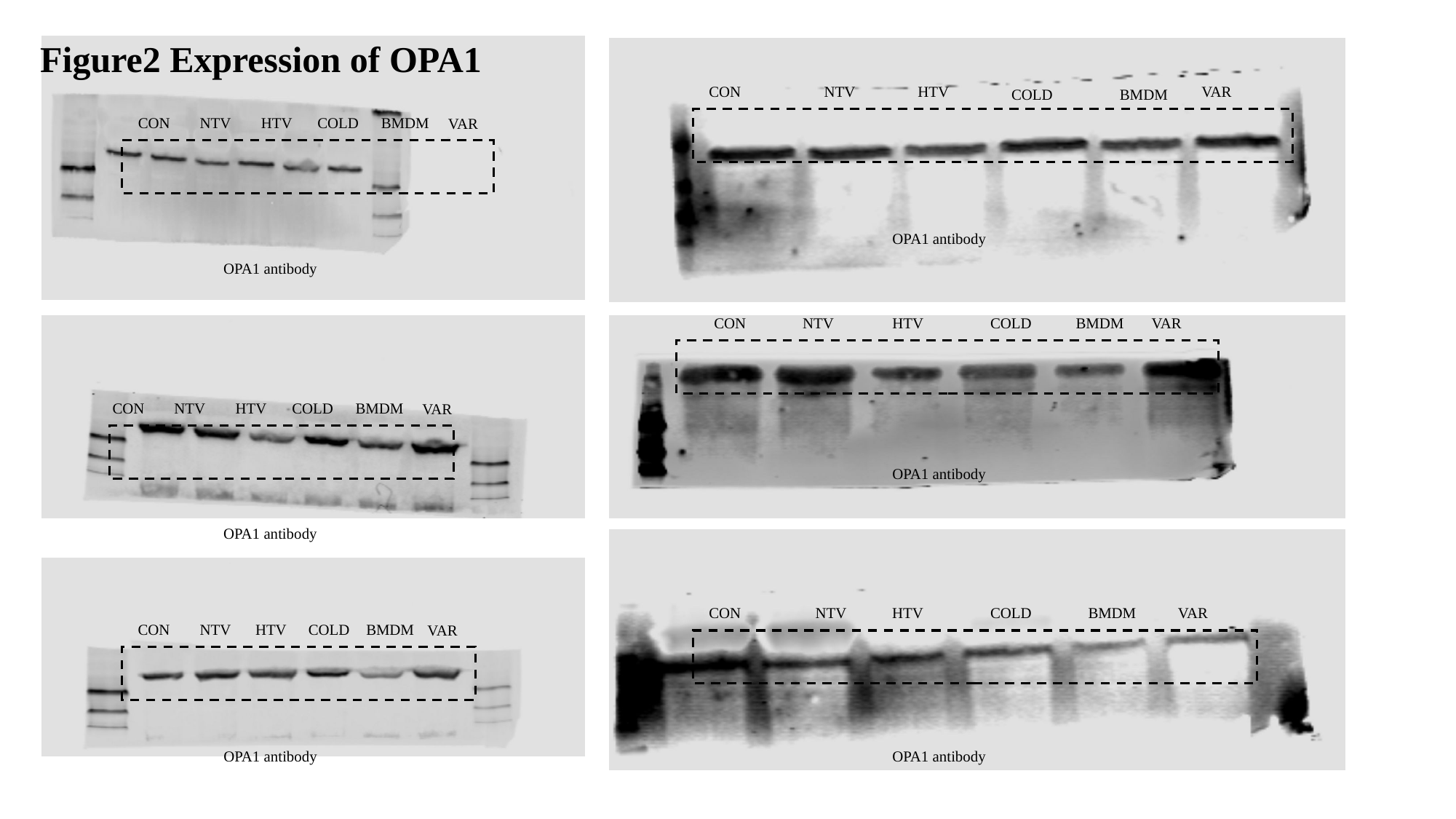

Figure2 Expression of OPA1
CON
NTV
HTV
COLD
BMDM
VAR
OPA1 antibody
OPA1 antibody
OPA1 antibody
OPA1 antibody
OPA1 antibody
OPA1 antibody
CON
NTV
HTV
VAR
COLD
BMDM
CON
NTV
HTV
COLD
BMDM
VAR
CON
NTV
HTV
COLD
BMDM
VAR
CON
NTV
HTV
COLD
BMDM
VAR
CON
NTV
HTV
COLD
BMDM
VAR

## Slide 3
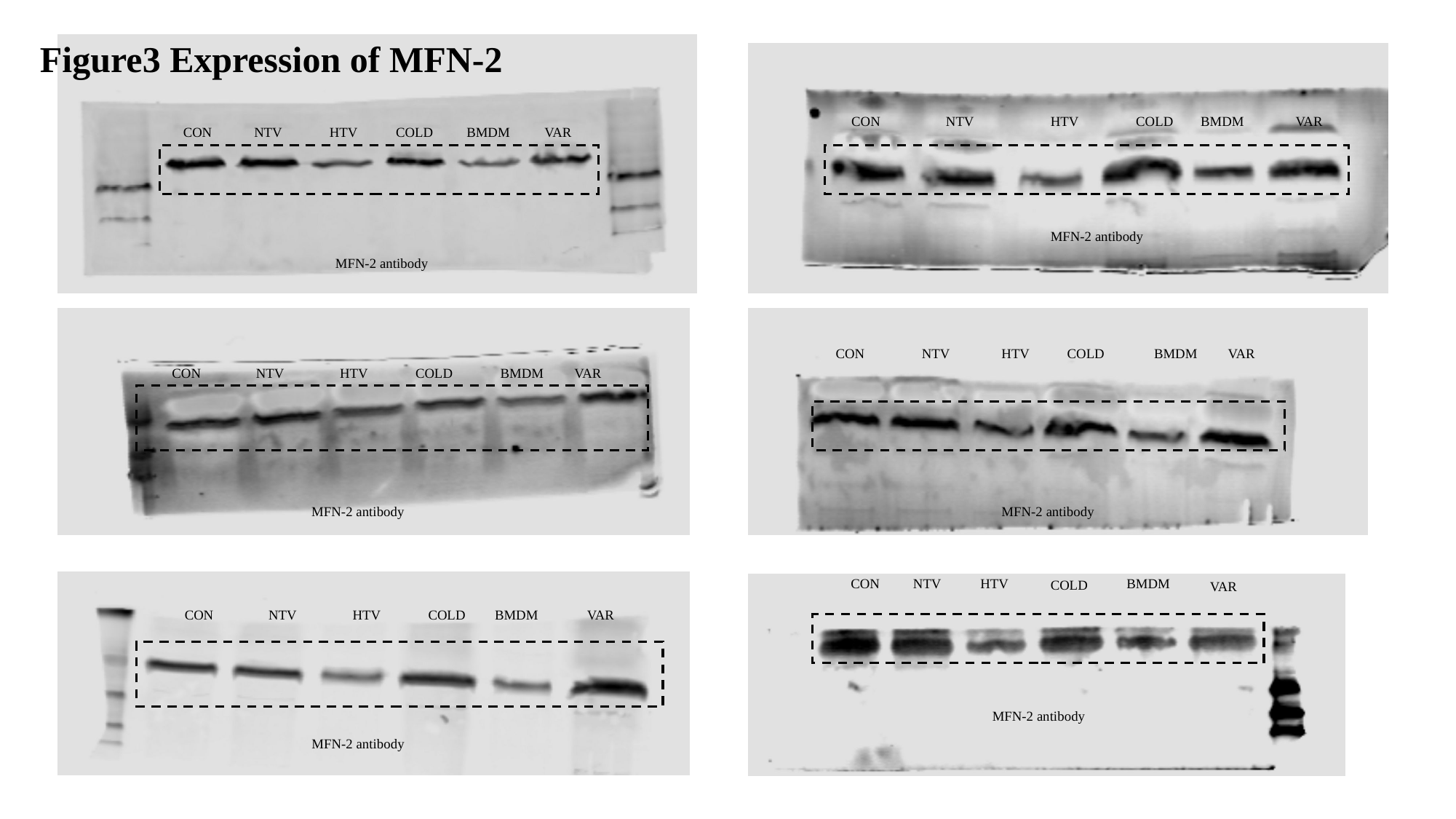

Figure3 Expression of MFN-2
CON
NTV
HTV
COLD
BMDM
VAR
CON
NTV
HTV
COLD
BMDM
VAR
MFN-2 antibody
CON
NTV
HTV
COLD
BMDM
VAR
CON
NTV
HTV
COLD
BMDM
VAR
CON
NTV
HTV
COLD
BMDM
VAR
MFN-2 antibody
MFN-2 antibody
MFN-2 antibody
CON
NTV
HTV
BMDM
COLD
VAR
MFN-2 antibody
MFN-2 antibody

## Slide 4
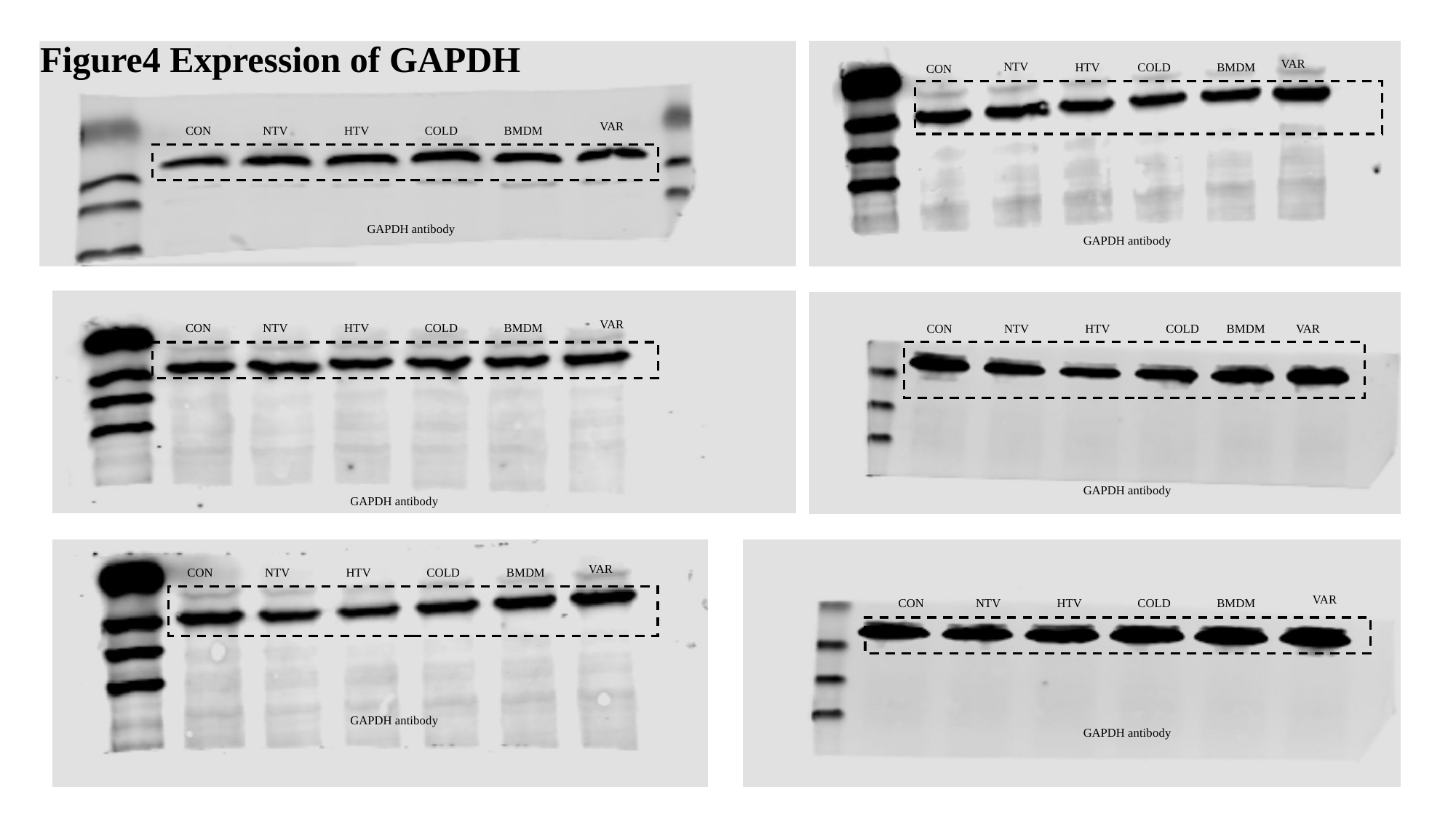

Figure4 Expression of GAPDH
VAR
CON
NTV
HTV
COLD
BMDM
GAPDH antibody
VAR
NTV
HTV
COLD
BMDM
CON
GAPDH antibody
VAR
CON
NTV
HTV
COLD
BMDM
VAR
CON
NTV
HTV
COLD
BMDM
GAPDH antibody
GAPDH antibody
VAR
CON
NTV
HTV
COLD
BMDM
VAR
CON
NTV
HTV
COLD
BMDM
GAPDH antibody
GAPDH antibody

## Slide 5
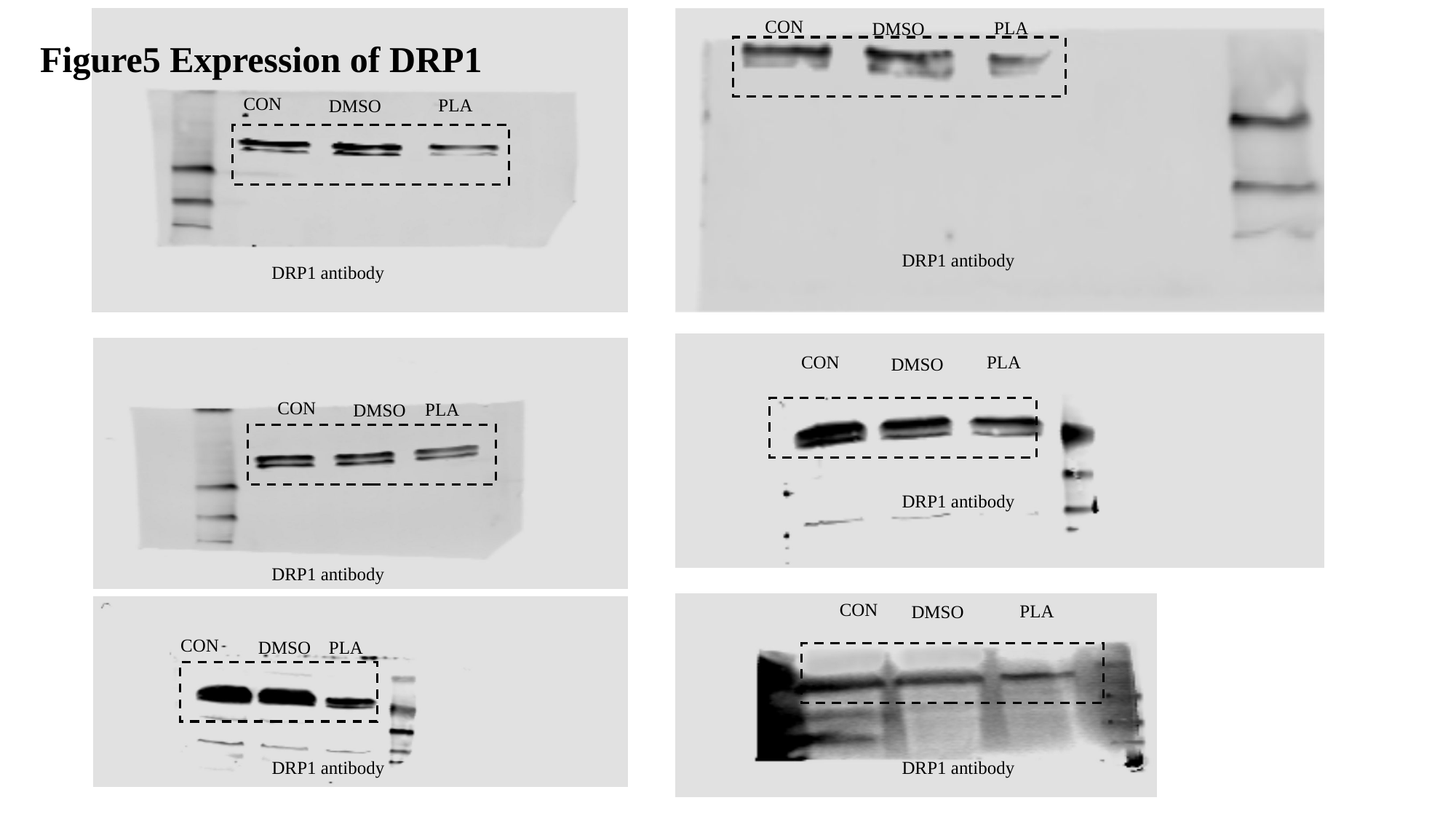

CON
PLA
DMSO
DRP1 antibody
DRP1 antibody
DRP1 antibody
DRP1 antibody
DRP1 antibody
CON
PLA
DMSO
Figure5 Expression of DRP1
DRP1 antibody
CON
PLA
DMSO
CON
PLA
DMSO
CON
PLA
DMSO
CON
PLA
DMSO

## Slide 6
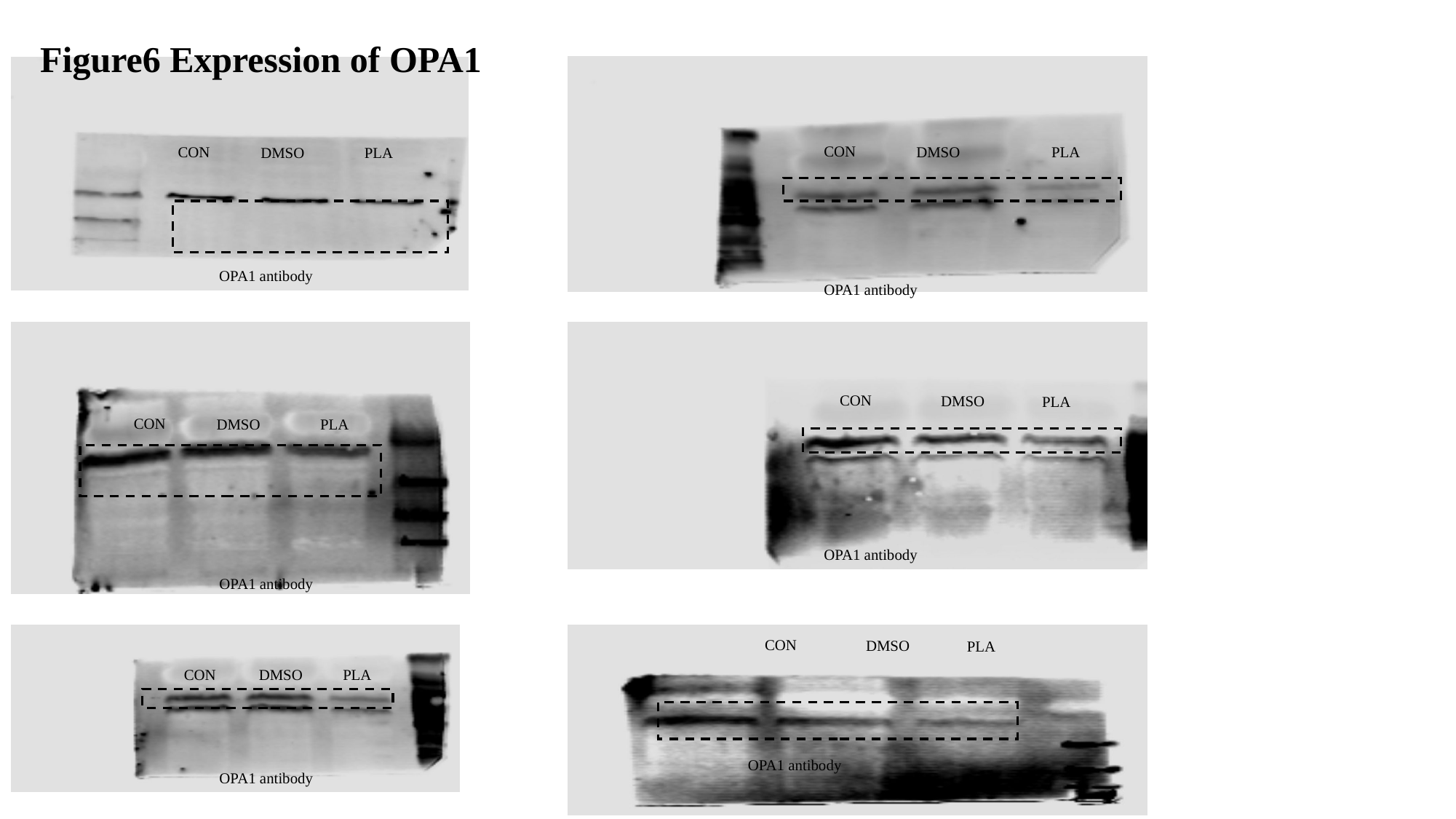

Figure6 Expression of OPA1
CON
CON
DMSO
PLA
DMSO
PLA
OPA1 antibody
OPA1 antibody
CON
DMSO
PLA
CON
DMSO
PLA
OPA1 antibody
OPA1 antibody
CON
DMSO
PLA
PLA
CON
DMSO
OPA1 antibody
OPA1 antibody

## Slide 7
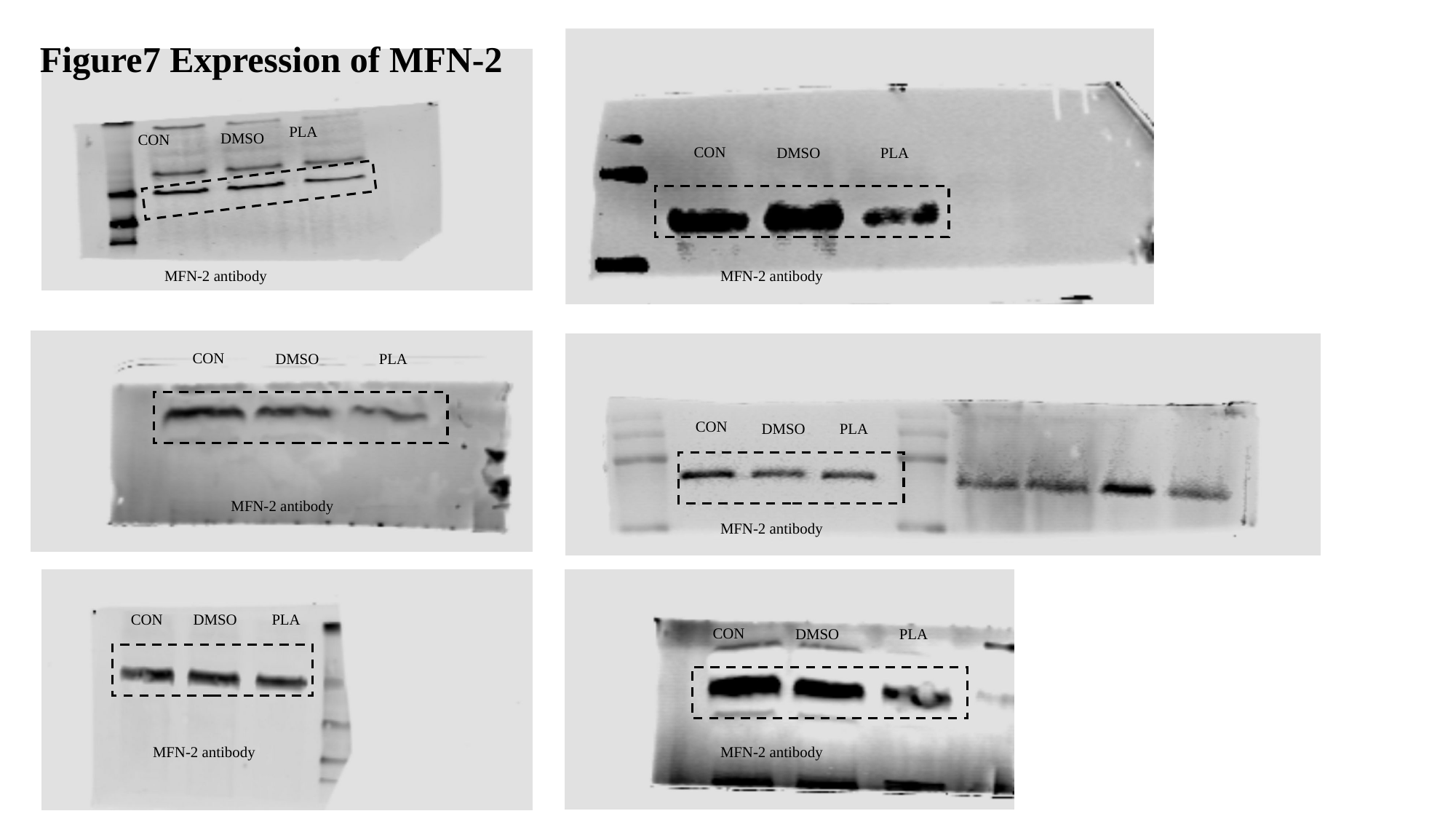

Figure7 Expression of MFN-2
PLA
DMSO
CON
CON
DMSO
PLA
MFN-2 antibody
MFN-2 antibody
MFN-2 antibody
MFN-2 antibody
MFN-2 antibody
CON
DMSO
PLA
CON
DMSO
PLA
MFN-2 antibody
PLA
CON
DMSO
CON
DMSO
PLA

## Slide 8
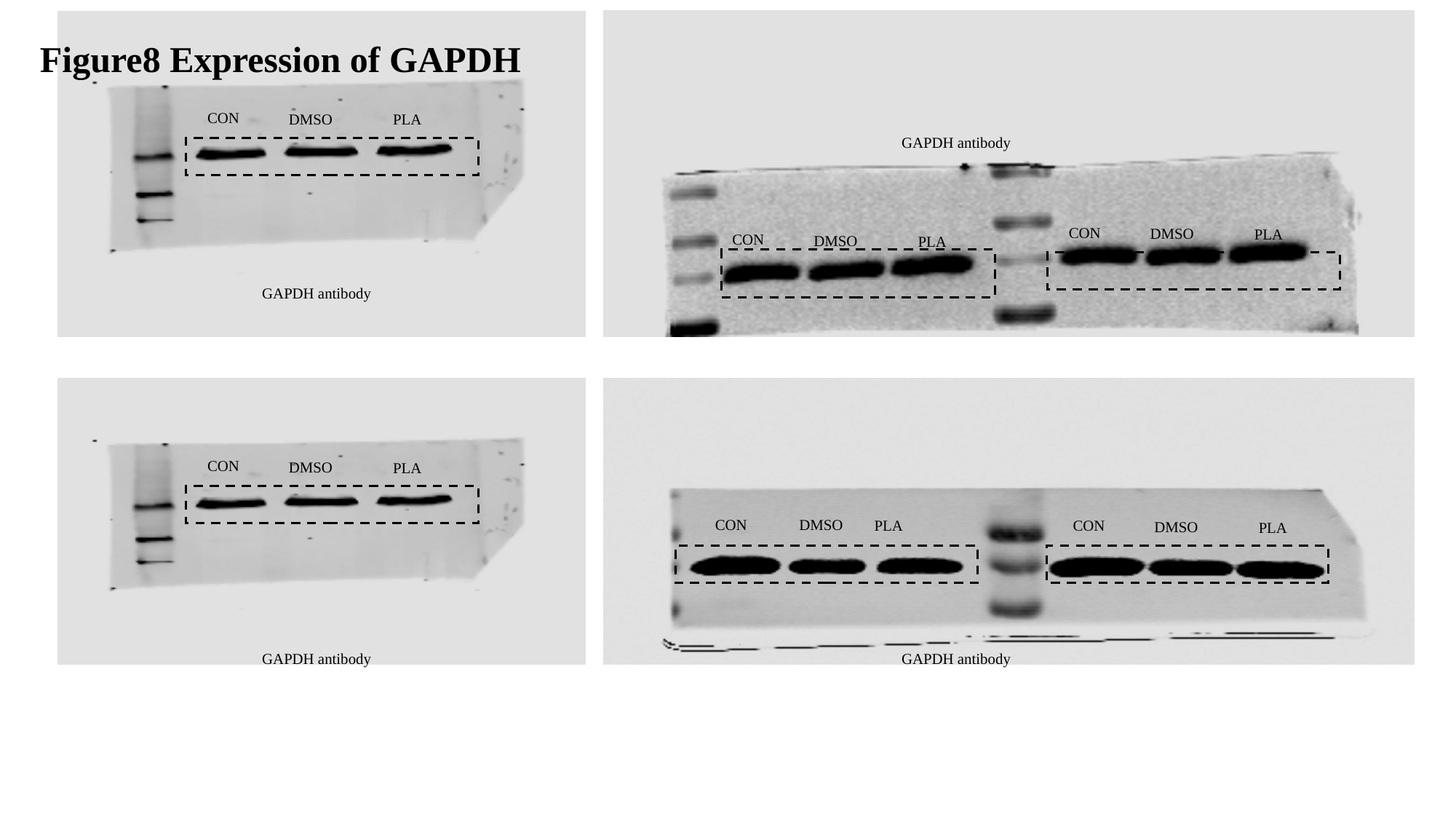

CON
DMSO
PLA
GAPDH antibody
GAPDH antibody
GAPDH antibody
GAPDH antibody
Figure8 Expression of GAPDH
CON
DMSO
PLA
CON
DMSO
PLA
CON
DMSO
PLA
CON
DMSO
PLA
CON
DMSO
PLA
